# Radiosurgically Treated Recurrent Cerebellar Hemangioblastoma: A Case Report and Literature Review

**DOI:** 10.3390/curroncol31070293

**Published:** 2024-07-09

**Authors:** François Fabi, Ève Chamberland, Myreille D’Astous, Karine Michaud, Martin Côté, Isabelle Thibault

**Affiliations:** 1 Service de Radio-Oncologie du Département de Médecine Spécialisée, Centre Intégré de Cancérologie (CIC), Hôpital de l’Enfant-Jésus, Centre Hospitalier Universitaire (CHU) de Québec-Université Laval, Quebec City, QC G1J 1Z4, Canada; francois.fabi@mail.mcgill.ca; 2 Service de Physique Médicale et de Radioprotection, CHU de Québec-Université Laval, Quebec City, QC G1J 1Z4, Canada; 3 Service de Neurochirurgie du Département de Chirurgie, Hôpital de l’Enfant-Jésus, CHU de Québec-Université Laval, Quebec City, QC G1J 1Z4, Canada

**Keywords:** hemangioblastoma, radiosurgery, radiation oncology

## Abstract

Background: Cystic, sporadic hemangioblastomas (HBLs) represent a unique, therapeutically challenging subset of central nervous system tumors, mainly due to their unpredictable growth patterns and potential for symptomatic progression. This study aims to explore the complexities surrounding the diagnosis, treatment, and long-term management of these lesions. Methods: A comprehensive literature review was performed, and a detailed case study of a 56-year-old patient with a cystic, sporadic cerebellar HBL was produced. Results: The case highlights the multiphasic growth pattern typical of cystic, sporadic HBLs, characterized by periods of dormancy and subsequent rapid expansion. An initial surgical intervention offered temporary control. Tumor recurrence, mainly through cystic enlargement, was treated by SRS. A subsequent recurrence, again caused by cystic growth, eventually led to the patient’s death. The intricacies of treatment modalities, focusing on the transition from surgical resection to stereotactic radiosurgery (SRS) upon recurrence, are discussed. Parameters indicating impending tumor growth, coupled with symptomatic advances, are also explored. Conclusions: The management of cystic, sporadic cerebellar HBLs requires a strategic approach that can be informed by radiological characteristics and tumoral behavior. This study underscores the importance of a proactive, individualized management plan and suggests guidelines that could inform clinical decision making.

## 1. Introduction

Hemangioblastomas (HBLs) are benign, highly vascular tumors predominantly found in the central nervous system. They are infrequent tumors, representing about 2% of intracranial neoplasia [1], and generally present as well-circumscribed, solid, and cystic masses composed of a rich capillary network embedded in neoplastic stroma, enveloped in a thin capsular layer. These lesions are classified as a World Health Organization (WHO) grade 1 neoplasm [2].

Sporadic HBLs are the most common type, representing approximately 70% of cases [1,3]. In that context, the presenting lesions are generally solitary, larger, and manifest in the 4th decade of life. However, most available longitudinal data emerge from the study of patients harboring von Hippel–Lindau (VHL) disease, caused by the germline mutation of the VHL gene. These lesions are, when compared with their sporadic counterparts, smaller, multifocal, and present earlier, generally at the third decade of life. Interestingly, sporadic cases harbor a somatic inactivation of VHL disease at a high frequency, with recent investigations reporting such alterations at rates of up to 76% [4]; alternatively, epigenetic inactivation of VHL disease also appears to be a driving mechanism of sporadic HBL tumorigenesis [5]. Germline and sporadic tumors are, once established, histologically indistinguishable from each other, further underlining their potentially shared underlying molecular etiology and subsequent response to treatment [6]. This partly drives the idea that, while their natural histories differ, the management of both entities should be comparable. 

Patients with VHL disease will develop craniospinal HBLs at a lifetime rate of 60 to 80%, with the cerebellum being the primary site of incidence in approximately 45% to 50% of cases, followed by spinal cord and brainstem, with respective incidences of 40% and 10% [6]. A recent review of the US National Cancer Database suggests a cerebellar incidence as high as 71.2%, further underlining the clinical significance of this specific anatomical presentation [7].

The main growth pattern for HBL is described as ‘saltatory’ growth that is characterized by periods of quiescence (2.1 ± 1.6 years) and interspersed periods of rapid growth (1.1 ± 1.3 years). Heterogeneity exists, featuring linear and exponential growth patterns rather than biphasic patterns, which are also associated with VHL disease [8]. While benign, these lesions can become severely morbid and potentially lethal. Symptoms emerge primarily due to the mass effect caused by the primary tumor as well as the development of peritumoral cystic lesions and pericystic edema, which are present in an estimated 70% to 75% of cerebellar symptomatic tumors [9,10]. Lesions in the cerebellum can also create hydrocephalus. These cystic lesions are more common in the cerebellum than any other CNS location and are strong predictor of symptoms. Compared with VHL-associated tumors, sporadic lesions are generally larger and are more often accompanied by a cystic component [11]. There is a wide degree of heterogeneity in the reported frequency of associated symptomology, with reported rates of headaches (12–75%), gait ataxia (12–64%), dysmetria (29–64%), and nausea/vomiting(8–34%) being highly heterogenous [9,12,13]. Interestingly, these tumors are seldom, although uniquely, associated with paraneoplastic polycythemia [14].

Imaging is the primary diagnostic modality used to identify HBLs. Because of the generally indolent and saltatory course of this disease, long-term screening is required, and a short lapse of non-recurrence is not indicative of future control. Given their highly variable growth trajectory, radiographic progression is generally not considered an implicit indication for treatment, either by surgery or stereotactic radiosurgery (SRS). However, identifying lesions bound to become symptomatic and suitable for early intervention remains challenging [6,12,15].

When treatment is indicated, surgical management is considered the standard of care and is historically considered to be curative in VHL patients [6]. This is not the case for sporadic hemangioblastomas, which display excellent short term local control but a retreatment rate of up to 40% [11]. On the other hand, radiotherapy is generally indicated for difficult-to-resect or residual disease cases. Although conventional fractionation regimen has historically been the primary option for the radiotherapeutic management of HBLs, SRS is now considered the gold standard for lesions under 3 cm [16]. Pooled analyses have shown SRS to be well tolerated, with a median adverse event rate of 3.1%, most commonly consisting of hydrocephalus, peritumoral edema, radiation necrosis, nausea/vomiting, and headaches [16]. While available data are highly variable, SRS generally demonstrates excellent local control for both VHL and sporadic lesions with a pooled 5-year PFS of 88.43% [16]. There is, however, a paucity of data on the effect of SRS on long-term control, especially in the context of sporadic HBLs. The long-term effectiveness of SRS in sporadic HBLs as well as the identification of the most appropriate candidates for its use demand further investigations.

In this article, we report our experience with a previously resected sporadic, cerebellar HBL with cystic components, which was treated at recurrence with SRS. Unfortunately, the patient subsequently relapsed again and passed away. We discuss early treatment indications for such lesions and present data supporting clinical decision making for their management.

## 2. Case Presentation

The patient, at presentation, was a 56-year-old male. He had no relevant past medical history and was overall healthy, with no allergies nor regular medications noted at the time of diagnosis and a smoking history of approximately 25 pack years. At the time, he worked in an industrial setting in which he operated heavy machinery. No family history appeared to be suggestive of VHL disease. The patient had reported a 2-week history of worsening balance issues, which made ambulating difficult. He denied B symptoms, nausea and vomiting, headaches, changes in vision, seizures, and any loss of consciousness. Upon physical examination, the patient was alert and oriented. His pupils were equal and reactive to light. Right-to-left nystagmus was found. Speech was normal and fluent. Light dysmetria was noted, which was worse on the right compared with the left. Power testing and reflexes were normal. The Romberg test was positive. The patient’s gait was noted as slightly widened.

A magnetic resonance imaging (MRI) analysis identified a cerebellar lesion in line with a hemangioblastoma. He was then transferred to our institution, a tertiary care center. A CT of the thorax, abdomen, and pelvis showed no other lesions. Abdominal ultrasound was performed to rule out renal abnormalities. Initial surgery to remove the right-sided posterior fossa large hemangioblastoma was performed in April 2006. The patient underwent a right suboccipital craniotomy. The tumor was localized in the right cerebellar hemisphere, starting from the lateral wall of the fourth ventricle with an extension almost reaching the right jugular foramen. Because of the highly vascular nature of this tumor, dissection around the tumor in the cerebellar was conducted. Arterial supply to the tumor was coagulated and cut. During dissection, the cystic part of the lesion was drained during the approach. After vascular supply was controlled, the tumor was “en bloc”. The pathology report documented a 20 × 10 × 3 mm tumor displaying a rich capillary network. The cells were positive for CD34, with peripheral cells being positive for glial fibrillary acidic protein (GFAP) and synaptophysin. The surrounding stroma was positive for vimentin; no cells were positive for keratin 8/18. MIB-1 was low. The findings were fully in line with an HBL rather than a metastatic deposit. Molecular analyses were performed on the patient’s blood. VHL sequencing was devoid of abnormalities, as was the multiplex ligation-dependent probe amplification assay, ruling out large deletions or amplifications. Taken together, these findings strongly supported a sporadic case of HBL.

On the post-surgical MRI, the surgical bed was described as a deep, right-sided, cerebellar 2 cm T1 hypo-intense irregular zone. Punctate 4–5 mm hypointense foci bordering the lateral aspect of the surgical site were noted, which were interpreted as a likely small hemorrhagic focus caused by a retained vascular element post surgery. The radiologist noted that the presence of a small tumoral residue could not be ruled out, but also that the lesion aspect was not in line with any cystic component. No hydrocephaly and a minimal pneumocephalus at the left anterior frontal region were noted. 

The post-operative course was uneventful, and the patient was discharged home on post-op day 4. Overall recovery was excellent, with normal motor and cognitive functions noted at 4 months post surgery. Yearly follow-ups were arranged for the first three years, which noted no sequalae and normal functions. The patient was henceforth seen every two years, with no symptomatic evidence of disease recurrence. 

**Figure 1:**
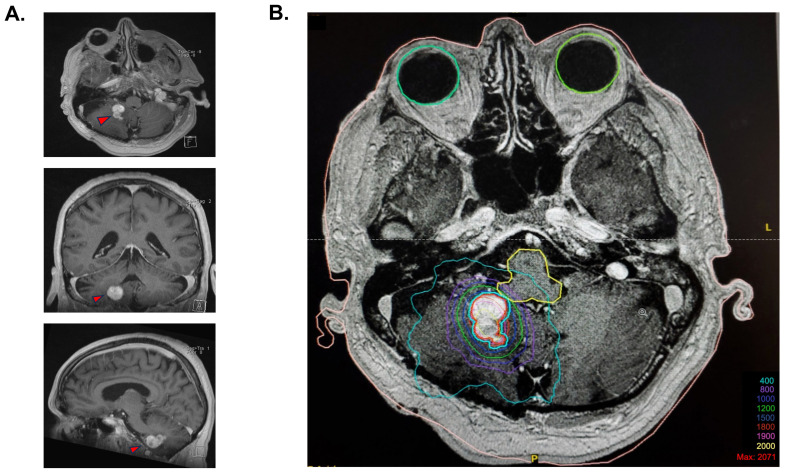
Stereotaxic radiosurgery planification. (**A**) Cerebral MRI T1 images prior to stereotactic radiosurgery, with a demonstration of the polylobulated lesion in the axial view, coronal view, and sagittal view. (**B**) Isodose coverage of the GTV (in red) for the 18 Gy radiosurgery plan. The blue contour is an expansion of 1 mm of the GTV treated at an 87% isodose because of the proximity with the brainstem. A total of 83% of the tumor volume was covered by the 18 Gy isodose, and 99.9% was covered by the 15 Gy isodose.

In 2007, a 2 cm progressing enhancing lesion was found, which was stable throughout 2008 and 2009. Minimal progression was again noted in 2010 and 2012. In September 2014, the enhancing portion of the lesion was noted as progressing from 19 × 12 × 15 mm to 20 × 15 × 16 mm (anteroposterior, transverse, cranio-caudal, respectively). A de novo circular, fluid-filled lesion was also noted anteriorly, measuring 8.1 × 15.2 × 13 mm. At the time of radiosurgery planning, the overall lesion measured 25 × 15 mm (AP, T, respectively), with the presence of solid as well as fluid components (Figure 1A). 

Radiosurgery for the slowly progressing lesion was recommended at the neuro-oncology tumor board, primarily because of the deep location of the lesion and its relapsing nature. The procedure took place on 17 December 2014. Immobilization was performed using a stereotactic frame, and an axial MRI T1 post-gadolinium sequence was fused to the planning CT scan for delineation of the target. A dosimetric plan was obtained using the X-Knife treatment planning system. SRS was delivered on a Varian Trilogy unit equipped with a high-definition multi-leaf collimator. The patient received an SRS of 18 Gy, which was prescribed at the 87% isodose, considering that the lesion was situated at millimetric distance to the brainstem to optimize the dose fall-off (Figure 1B). A total of 83% of the tumor volume was covered by the 18 Gy isodose, and 99.9% was covered by the 15 Gy isodose. The gross tumor volume (GTV) was 3.77 cm^3^. The treatment volume ratio (TVR) was 1.2, and the prescription isodose to target volume (PITV) was 1.0. The maximal point dose to the brainstem was 13.1 Gy. The volume of normal brain that received 12 Gy was 7.9 cm^3^. 

The patient reported headaches 1 to 2 months post SRS, which were well controlled with acetaminophen. He was otherwise asymptomatic and experienced no adverse effects. The first follow-up MRI at 3 months post-op showed mild peripheral enhancement of the lesion that was likely caused by radiation necrosis, which caused a slight mass effect on the cerebellar peduncle. The patient was asymptomatic at the time. 

**Figure 2:**
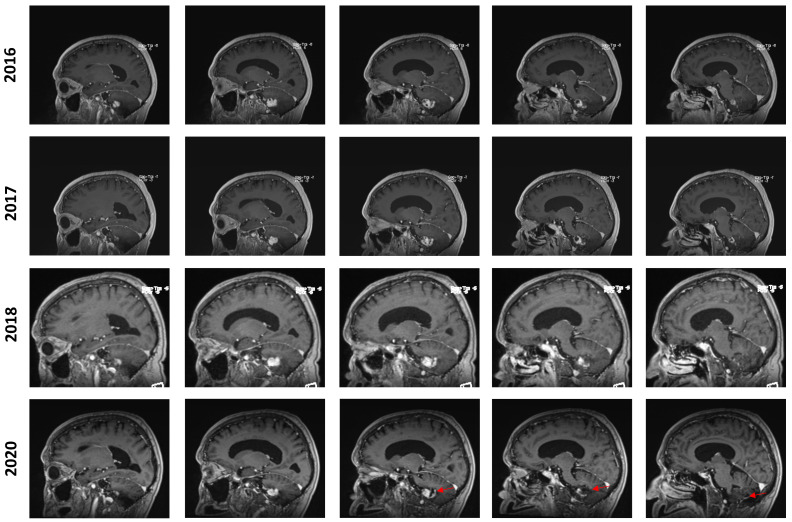
Longitudinal follow-up. Representative capture of sagittal T1-weighted MRI control imaging. The date of imaging is represented. Images are organized left to right and represent a left–right progression in the sagittal plane. The red marker highlights the appearance of an enlarging cystic component in the posterior aspect of the lesion in 2020.

In July 2016, an MRI showed a mild increase in the lesion’s size from 24 × 17 × 17 mm to 26 × 20 × 17 mm (AP, T, CC, respectively). In the yearly imaging follow-ups, the lesion was found to be slowly shrinking, reaching a size of 18 × 13 × 14 mm in September 2020. However, at this time, the lesion still displayed a prominent cystic component that appeared to be slightly progressive. In retrospect, it appears that the solid component of the lesion remained stable while the cystic component was discreetly progressing, especially from 2018 to 2020 (Figure 2). No new lesions were found during the clinical course of the patient.

**Figure 3:**
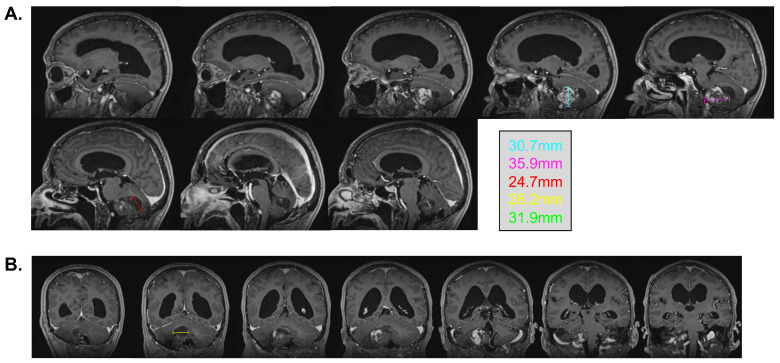
Pre-surgical MRI of the lesion. Representative capture of the T1-weighted MRI pre-surgical imaging. Representative measurements are color-coded. (**A**) Sagittal images are organized left to right and represent a left–right progression in the sagittal plane. (**B**) Coronal images are organized left to right and represent a posterior-to-anterior progression in the coronal plane.

**Figure 4:**
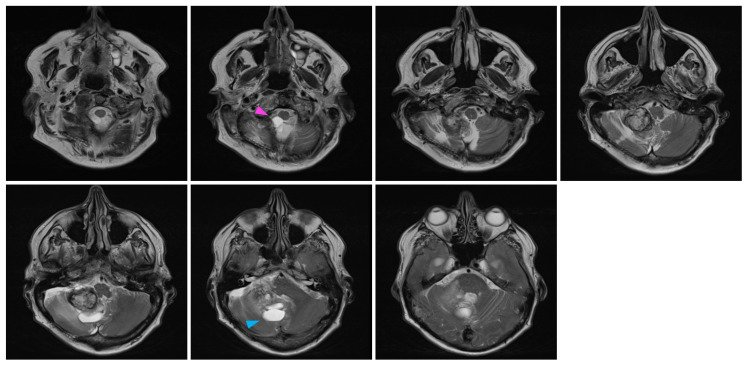
Pre-surgical MRI of the lesion. Representative capture of the T2-weighted MRI pre-surgical imaging highlighting the two cystic components of the lesion. Coronal images are organized left to right and represent a posterior-to-anterior progression in the coronal plane. The pink marker highlights a 21 mm antero-infero-medial cystic lesion, while the blue marker highlights a 32 mm posterior cystic lesion.

In February 2022, at the age of 72 years old, the patient was hospitalized in his hometown following the appearance of symptoms and was referred to neurosurgery. He mainly displayed loss of balance, unexplained loss of weight, confusion, and psychomotor retardation. He was able to ambulate without the aid of a walker, albeit at a very slow pace. Dexamethasone failed to improve his condition. An MRI underlined a right-sided, cerebellar intra-axial lesion located at the anterolateral portion of the cerebellar hemisphere. The lesion, determined to be a relapse of the initial HBL, displayed a nodular component in intimate proximity with the brainstem, measuring 36.2 × 32.1 × 31.9 mm (Figure 3). The presence of two cystic components were also noted. The first, located posteriorly, measured 32 mm maximally (Figure 4, blue marker). The second, located antero-infero-medially, measured 21 mm maximally and protruded in the subarachnoid space at the level of the pons (Figure 4, pink marker). The lesions caused a mass effect on the medulla, the inferior aspect of the pons, the middle cerebellar peduncle, and at the level of the 4th ventricle with associated hydrocephalus.

The patient was transferred to our institution for neurosurgical intervention. He underwent a sub-occipital craniotomy and hemangioblastoma resection in March of 2022. The pathology confirmed an HBL. A post-operative MRI noted a 98–99% surgical resection of the lesion, with the remaining nodular residue grafted to a 3 cm ischemic zone at the posterior cerebellum. A 3 cm longitudinal sinus vein thrombosis was also noted at a distance from the operative site. The hydrocephalus was slightly reduced. 

The post-operative course of the patient was complicated. He was initially admitted to the ICU and was extubated two days later. He was eventually transferred to floor 7 days later. While stable, he was noted as delirious and confused, having difficulty communicating, dysphagia, and an inability to ambulate due to weakness and severe balance impairment. Two days later, the sinus vein thrombus was noted to have been resolved. The hydrocephalus remained generally unchanged from the previous state. This prompted a therapeutic evacuation of cerebrospinal fluid, and a lumbar puncture culture demonstrated that the patient had developed S. warneri meningitis. Efforts at therapeutic cerebrospinal fluid evacuation and treatment for meningitis using vancomycin did not improve the patient’s motor or cognitive function. Rehabilitation measures were largely ineffective, and the patient’s condition declined, leading to palliative care and his eventual passing in early May of 2022.

## 3. Discussion

This case highlights the challenges of the long-term management of a patient presenting a cerebellar, sporadic HBL with cystic features and adds to the limited literature focusing on long-term outcomes of the radiosurgical treatment of such lesions. In the presented case, the patient’s lesion demonstrated a multiphasic growth pattern, with long periods of quiescence interspersed by volume progression. The initial surgical excision for a symptomatic tumor allowed for local control for approximately 8 years. The asymptomatic radiological progression of the lesion prompted radiosurgical intervention. SRS provided local control again for approximately 8 years until a severely symptomatic lesion recurrence prompted a second surgical excision. Unfortunately, the patient clinical course was complicated by hydrocephalus and S. Warneri meningitis. The overall clinical trajectory led the family to elect for the patient to be oriented towards a palliative care approach, and he eventually passed.

Surgical excision provides immediate symptomatic relief in eloquent lesions and exhibits near-perfect local control when total resection is achieved, with a relapse rate of 0%, even in studies with decades of follow-up [12,17]. Sporadic lesions, however, display a different outcome profile. A recent extended follow-up (median 62 months, range 1–347 months) study reported a retreatment rate of nearly 40% in the 116 sporadic cases they followed [11]. Given the lower long-term efficacy of surgical interventions in these lesions, sporadic HBLs could be particularly well suited for alternative, well-tolerated treatment modalities such as SRS.

There is also a need for more data regarding the role of SRS in treating HBLs; particularly evidence based on long-term follow-up. Pan and colleagues performed the largest systematic review and meta-analysis examining the use of SRS in CNS HBLs. Their cohort included both sporadic and VHL-related lesions and exhibited a pooled 5-year PFS of 88.44%. Unfortunately, considering the heterogeneity of the follow-up periods included in the study, PFS could not be computed for a longer interval. It is therefore difficult to expound on the appropriateness of SRS for long-term control of these tumors based on this review. However, studies with longer follow-up can provide additional information.

Smaller, retrospective studies using Gamma Knives have shown a favorable SRS effectiveness at controlling HBLs, with a local tumor control rate of 78–96% at 10 years being reported [18,19]. One of the rare prospective trials that examined SRS for HBLs in twenty VHL disease patients showed a local control of 91% at 2 years, 83% at 5 years, 61% at 10 years and 51% at 15 years. Their results suggest a reduction in local control as follow-up periods become longer [20].

Larger studies are also available and present slightly different results. Kano et al. conducted the largest retrospective multicenter international study examining SRS for the treatment of intracranial HBLs. They examined patients with VHL-associated HBLs (*n* = 80, 335 tumors) as well as sporadic tumors (*n* = 106, 182 tumors). The results showed a pooled PFS of 92%, 89%, and 79% at 3, 5, and 10 years, respectively, with the local control being slightly superior in VHL-associated tumors compared with sporadic lesions (82% vs. 75% at 10 years). Smaller tumor volume, solid tumor type, and higher marginal dose were also associated with better outcomes [21]. While marginal dose-dependent control has been suggested in previous investigations, this notion has been challenged by results from meta-analyses that failed to identify such a relationship [16,22]. Additionally, radiation necrosis appears to be correlated to marginal dose [16,19]. This underlines the delicate balance that must be achieved to reach maximal efficacy, as increased dose might improve outcomes but is at higher risk to cause side effects. In the particular case of our patient, we acknowledge that the marginal dose of 15 Gy, which was mainly limited due to the brainstem’s proximity, could be considered to be in the lower range of dosage. Another restriction of the dose coverage we achieved in this plan is essentially due to technical limitations. Indeed, this treatment was performed ten years ago in 2014. While 83% of the tumor received 18 Gy, a similar treatment performed today within the context of a SRS-specialized facility would certainly achieve a higher dose coverage while respecting brainstem dose constraints.

Regarding the efficacy of SRS for HBLs, similar results were reported in the retrospective study by Hanakita et al., which included SRS-treated patients with VHL (*n* = 14) and sporadic (*n* = 7) lesions, mostly in the cerebellum (86/97 total tumors). They reported a PFS of 92%, 92%, and 80% at 3, 5, and 10 years, respectively. However, the 10-year control rates were superior for VHL-associated HBLs (83%) when compared with sporadic HBLs (44%) [23]. The authors’ univariate analysis suggested that improved PFS was associated with lesions that were solid, small (<0.13 cm^3^), and VHL-associated. Interestingly, the median volume of VHL-associated lesions (0.1 cm^3^) was significantly smaller than sporadic lesions (2.9 cm^3^). This association between VHL disease and smaller tumor size is likely dependent on the increased radiological surveillance that these patients undergo. Given the underlying histological and molecular similitudes between sporadic and VHL-associated HBLs, the difference in outcomes is plausibly due to tumor volume at time of treatment and by the fact sporadic lesions harbor cystic components at a higher rate. Nonetheless, the results from these trials indicate that SRS presents good rates of long-term local control, with superior results being observed for smaller lesions. It thus seems appropriate to recommend SRS for the treatment of these tumors.

Nevertheless, there is still uncertainty on the role of treatment, either through surgical resection or SRS, of early asymptomatic lesions. Many groups recommend against intervening on asymptomatic HBLs, even in the presence of radiographic progression [6,15,16,20,21]. However, other reports, which were aimed at evaluating the long-term effectiveness of SRS, have concluded that early radiosurgical intervention is a potent, effective approach to control progressive, anatomically high-risk, asymptomatic lesions [23]. This uncertainty stems from the difficulty in identifying lesions that would benefit from intervention compared with observation, as many will remain symptomatically silent. The challenge thus lies in the proper identification of asymptomatic lesions that are bound to become problematic and warrant early treatment.

Growth kinetics have been suggested as a predictive tool for symptom emergence. In the case of cerebellar HBLs, Ammerman et al. found that a combined tumor/cyst presenting a growth rate of >122 mm^3^/month was predictive for eloquent lesions. In the case of more slowly progressive lesions, a combined absolute volume of >69 mm^3^ (equivalent to a sphere of 5.2 mm diameter) was also predictive. It should be noted that their algorithm was optimized to deliver a sensitivity of 100% and specificity of 72%, thus being biased towards the over-detection of future symptomatic lesions [15]. Other groups have examined cyst size as a predictor of symptom onset and identified a threshold of 20.3 mm as a predictor (specificity 90%) of appearance of symptoms for cerebellar HBLs [10]. This is particularly interesting given that tumor growth rate appears to be affected by the appearance of a cystic component in lesions. Indeed, in a retrospective analysis of 184 patients presenting VHL-associated and sporadic HBLs, the median growth rate of tumors was increased from 1.6 mm^3^/month to 82.4 mm^3^/month once a cystic component appeared in the tumor [11]. This strongly suggests that the appearance of a cystic component in a previously nodular tumor is a powerful indicator of impending volume progression and thus the symptom’s emergence.

Cerebellar HBLs are also generally the most likely intracranial HBLs to become symptomatic if left untreated, with rates of 70% at 10 years being reported in natural history studies [15]. Moreover, it appears that cerebellar HBLs presenting a cystic component are likely to progress faster, with the growth rate being the highest in the cerebellum when compared with the brainstem (5.8-fold) and spinal cord (4.6-fold) [10]. These data support the idea that cerebellar HBLs could represent a particularly relevant early treatment target as they are distinctively poised to become symptomatic.

Taken together, these data support a proactive approach in which SRS could be used on small, sporadic cerebellar HBLs. These lesions appear to be intrinsically more prone to rapid and symptomatic progression, and their explosive growth can be heralded by the emergence of a cyst in a previously solid lesion. Furthermore, sporadic lesions are more likely to develop a cystic component than VHL-associated lesions, increasing their intrinsic potential for progression. Because smaller lesions would be treated, this strategy also presumably allows for a heightened marginal dosage with limited risk for adverse effects, with possible improvement in local control. Treatment could be considered as soon as disease recurrence or radiological progression is observed, particularly in unfavorable surgical candidates. Lesions that reach the suggested threshold of 20.3 mm or progress at a rate of >122 mm^3^/month could warrant closer follow-up and be considered for prompt treatment. In all cases, early asymptomatic progression post-SRS should be differentiated from radiation-induced pseudoprogression [23], a confounder that disappears as time from therapy extends.

Finally, it should be noted that most of the presented data pertain to the primary treatment of HBLs. Radiotherapy can be used in the adjuvant setting as well, which is represented in the most recent NCCN guidelines [24], which recommend that partially resected symptomatic lesions should undergo adjuvant radiotherapy. No specific modality is preferred, with reports of SRS, hypofractionation, and fractionated regimens being documented. In our center, we usually use single-fraction SRS for tumors less than 2.5 cm, while larger lesions are now treated with a five-fraction stereotactic radiation therapy on our dedicated radiosurgery unit using the same immobilization frame, micro-multileaf (microMLC), and six-degree-of-freedom (DOF) couch. As our SRS practice evolves and becomes more refined with time, we expect this modality to be central to the management of these patients. However, more data appear necessary to objectively determine the best treatment strategy.

## 4. Conclusions

It is our opinion that the available data support a robust role for SRS in the management of HBLs. Technological advances provide ever-improved dose control and anatomical precision, which translate into excellent outcomes with minimal side effects. Nevertheless, identifying lesions that would most benefit from SRS remains a challenge. We propose that asymptomatic, small, anatomically high-risk sporadic hemangioblastomas that progress and present features that are likely to cause symptoms to emerge are well-suited for SRS treatment with minimal risk of adverse outcomes. We believe that SRS is a potent tool in the management of these lesions, and such an option should be examined by the multidisciplinary management team when selecting the optimal treatment options for patients.

## Data Availability

Original images and radiotherapy protocols are available at the reader’s request.
